# Chronic cutaneous and mucosal mucormycosis: *Rhizopus arrhizus* as a major pathogenic fungus

**DOI:** 10.1080/22221751.2025.2477653

**Published:** 2025-03-07

**Authors:** Xingyu Li, Chang Miao, Jinlei Yu, Fang Liu, Zhenlai Zhu, Jixin Gao, Dong Yan, Luming Hai, Gang Wang, Yubo Ma, Yanyang Guo, Meng Fu

**Affiliations:** a Department of Dermatology, Xijing Hospital, Fourth Military Medical University, Xi’an, People’s Republic of China; b Department of Dermatology, Nanjing Drum Tower Hospital, The Affiliated Hospital of Nanjing University Medical School, Nanjing, People’s Republic of China; c State Key Laboratory of Oral & Maxillofacial Reconstruction and Regeneration, National Clinical Research Center for Oral Diseases, Shaanxi Key Laboratory of Stomatology, Department of Oral Medicine, School of Stomatology, Xi’an, People’s Republic of China

**Keywords:** Chronic cutaneous mucormycosis, mucorales, histopathology, mycology, clinical manifestations

## Abstract

Chronic cutaneous mucormycosis is a rare condition distinct from the acute form, characterized by a prolonged, indolent course and varied clinical presentations. This study presents a 5-year experience from a tertiary dermato-mycology clinic, identifying six cases, the majority of whom were immunocompetent, with trauma history reported in four patients. The median duration from symptom onset to diagnosis was 60 months. The primary pathogens identified were *Rhizopus arrhizus*, *Mucor variabilis*, and *Lichtheimia ramosa*. Histopathological analysis demonstrated the absence of fungal angioinvasion, a hallmark of acute mucormycosis, which likely accounts for the slower progression observed in chronic cases. Systemic Amphotericin B treatment achieved favourable outcomes in most patients though significant morbidity persisted in some cases. This case series underscores the clinical and pathological distinctions of chronic cutaneous mucormycosis, highlighting the potential influence of host factors and environmental conditions on chronicity. The predominance of *Rhizopus arrhizus* suggests that chronicity is driven more by hostpathogen interactions than fungal species-specific factors. Increased recognition of the atypical clinical features, such as diverse cutaneous manifestation and slower progression course, as well as the utilization of diagnostic tools including histopathology, fungal culture, and advanced molecular techniques, is essential for the timely diagnosis of this rare presentation.

## Introduction

Mucormycosis is a severe fungal infection caused by species belonging to the order Mucorales, with mortality rates ranging from 40% to 80%. Its incidence has been increasing due to the rising prevalence of diabetes, organ transplantation, chemotherapy, and immune-related medical conditions [1]. Among the causative agents, *Rhizopus* spp. is the most common genus in human mucormycosis cases, followed by *Mucor* spp. and *Lichtheimia* spp., which account for 70–80% of all mucormycosis infections [2]. Based on the site of infection, mucormycosis can be classified into six categories: rhino-orbital-cerebral mucormycosis (ROCM), pulmonary mucormycosis, cutaneous mucormycosis, gastrointestinal or renal mucormycosis, disseminated mucormycosis, and other uncommon focal sites mucormycosis [3]. Following ROCM and pulmonary types, cutaneous mucormycosis is the third most common clinical form and can be further subcategorized into three subtypes [4]. Patients with infections confined to the cutaneous or subcutaneous tissue are considered to have a localized disease. Patients with invasion into muscle, tendon or bone are classified as having a deep extension of infection. Finally, patients with cutaneous involvement alongside a non-contiguous site are defined as having disseminated infection.

Clinically, cutaneous mucormycosis typically presents with an acute onset and rapid progression. The histopathologic hallmark of acute cutaneous infection with Mucorales is extensive angioinvasion, which is accompanied by tissue necrosis. This feature is reflected in its various clinical manifestations, including targetoid plaques, bull’s-eye infarctions, ecchymotic lesions, and lesions with necrotic centers resembling ecthyma gangrenosum [5]. In rare cases, cutaneous mucormycosis may present as a chronic course of this infection [6]. Chronic cutaneous mucormycosis has been mainly reported for unusual Mucorales. Specifically, *Mucor (M.) irregularis* has been identified as a causative agent in chronic cutaneous infection documented in China [7], Japan [8], and India [9]. Due to its inconsistency with the classic description of the disease, chronic cutaneous mucormycosis often presents atypically, necessitating a diagnostic approach distinct from that of the acute form. Herein, we report on the clinicopathologic and mycologic characteristics of 6 patients diagnosed with chronic cutaneous and mucosal mucormycosis. Remarkably, our findings reveal that in addition to *M. irregularis*, commonly encountered species such as *Rhizopus (R.) arrhizus* (formerly *R. oryzae*) can cause this chronic form of infection. Histopathological analysis demonstrated fungal hyphae in all cases, yet none exhibited angioinvasion. As angioinvasion is typically regarded as the main factor in the rapid clinical progression of mucormycosis, its absence may underline the chronic course observed in cutaneous and mucosal manifestations. This suggests that the pathogenesis of chronic mucormycosis might be attributed to the lack of angioinvasion, indicating a need for specialized diagnostic and therapeutic strategies for managing such prolonged cases.

## Materials and methods

The database of the Department of Dermatology at Xijing Hospital, a tertiary university-affiliated hospital in China, was systematically reviewed to identify all patients diagnosed with cutaneous and mucosal mucormycosis who attended the dermato-mycology clinic between 2017 and 2024. Diagnosis criteria and therapeutic principles strictly adhered to the *Global Guideline for the Diagnosis and Management of Mucormycosis* [1] and *Expert consensus on diagnosis and management of mucormycosis in China (2022)* [10]. Chronicity was defined in alignment with ROCM standards [11], specifying a symptom duration of at least four weeks. Based on the above information, the diagnosis is confirmed through the following aspects: (1) Persistent cutaneous or mucosal lesions exceeding 4 weeks. (2) Histopathology shows broad, thin-walled, pauci-septate or aseptate hyphae in the dermis and subcutaneous tissue. (3) Fungal culture or tissue molecular biology identified the infection as mucorales. (4) Etiological examination, imaging examination, and biochemical examination are used to rule out other infections. Medical records of the eligible patients were examined to collect comprehensive data, including past medical history, clinical manifestations, histopathological findings, imaging findings, treatment procedures and prognostic outcomes. Mycological information, particularly etiological organisms, was also systematically documented.

All diagnoses, treatments, and follow-up assessments were conducted at the dermato-mycology clinic, with photographic documentation of the lesions undertaken for each patient. Histological and microbiological specimens were analyzed by the mycology laboratory technician and dermatopathologist, respectively. The slides were re-evaluated by a dermatopathologist from our department. Only cases with microbiologic evidence findings of Mucorales infections from deep tissue biopsy, and/or corresponding pathological findings indicating intradermal or subcutaneous Mucorales elements, confirmcutaneous Mucorale infection.

To summarize the patient characteristics, we utilized non-parametric statistical methods due to the small sample size and non-normal distribution of the data. Continuous variables are presented as medians with interquartile ranges (IQR), while categorical variables are expressed as frequencies and percentages. The median duration from symptom onset to diagnosis was calculated, and the range of patient ages at presentation was determined.

## Results

**Figure 1.**
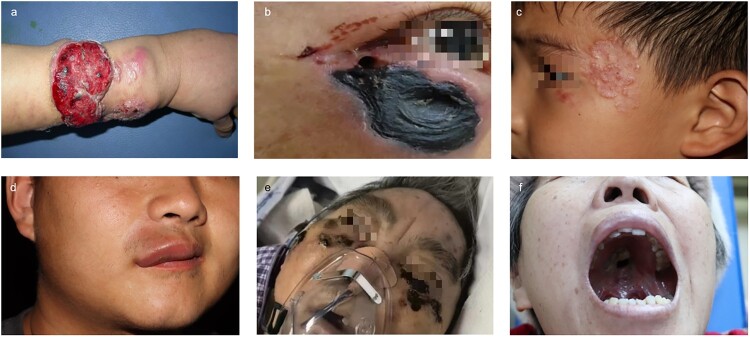
Cutaneous and mucosal manifestations in 6 cases of mucormycosis patients. (a)Three visible masses are seen on the right forearm of Patient 1. Some are vegetative and accompanied by surface erosion. (b) Necrotic ulcers with black crust are seen on the periorbital region of the right eye of Patient 2. (c) Slightly elevated circinate plaques are seen on the left temporal side in Patient 3. (d) Indurated swelling on the right upper lip in Patient 4. (e) Several necrotic plaques are seen on bilateral malar areas in Patient 5. (f) Necrotic ulcer on the soft palate in Patient 6.

Six patients with histologically and/or microbiologically confirmed chronic cutaneous and mucosal mucormycosis were identified, with their characteristics summarized in Table 1. The male-to-female ratio was 2:1, and the distribution of age was characterized by a median of 44 and quartiles at Q1 22 and Q3 57 (range 5–75 years). All patients exhibited a chronic disease course, with the interval between lesion onset and diagnosis ranging from 1 to 120 months, and the median is 5 months, with quartiles dividing the data into four parts: Q1 1.75 months and Q3 48 months. One patient (Patient 1) showed lesions on the right forearm, presenting multiple confluent nodular lesions with surface erosions (Figure 1(a)). Four patients had facial involvement: Patient 2 exhibited necrotic ulcers with black crusts on the periorbital region of the right eye (Figure 1(b)), which eventually progressed to vision loss and scleral rupture. Patient 3 presented with slightly elevated, circinate plaques on the left temporal area (Figure 1(c)). Patient 4 showed indurated swelling on the right upper lip (Figure 1(d)), and Patient 5 had necrotic plaques on bilateral malar areas (Figure 1(e)). The remaining patient, Patient 6, displayed a necrotic ulcer on the soft palate of the oral mucosa (Figure 1(f)).

**Table 1.** Clinical data for the six patients with chronic cutaneous mucormycosis.

Patient	Age at onset(Y)	Sex	Underlying condition	Location	Pathogenetic methods undertaken	Imagological examinations	Causative Fungi (GenBank No.)	Therapeutic drugs	Surgery undertaken	Outcomes at last follow-up
1	40	Male	Trauma	Right forearm	Histopathology	None	Not speciated	AmB-D	None	Survive without sequelae
2	49	Male	Trauma	The periorbital region of the right eye	Histopathology mNGS analysis FISH	CT MRI	*L. ramose* (PQ805236)	AmB-D Itraconazole	Right eye exenteration	Survive with sequelae (eyeball excision)
3	5	Male	Trauma	Left temporal	Histopathology Fungal culture ITS sequencing analysis	None	*R. arrhizus* (PQ804040)	L-AmB	None	Survive without sequelae
4	28	Male	Hyperlipidemia Trauma	Right upper lip	Histopathology Fungal culture ITS sequencing analysis	CT MRI	*R. arrhizus* (PQ849241)	ABCD Itraconazole	None	Survive without sequelae
5	75	Female	Type II Diabetes Trauma	Bilateral malar areas	Histopathology Fungal culture ITS sequencing analysis	Ultrasonography	*R. arrhizus* (PQ870347)	None	None	Death
6	51	Female	Stomach Cancer	Soft palate Paranasal sinus	Histopathology Fungal culture ITS sequencing analysis	CT MRI Electronic rhinoscopy Ultrasonography	*M. variabilis* (PQ805236)	L-AmB Itraconazole	None	Survive with sequelae (Palatal perforation)

ABCD Amphotericin B Colloidal Dispersion, AmB-D Amphotericin B deoxycholate, CT Computed Tomography, ITS internal transcribed spacer, L-AmB Liposomal Amphotericin B, mNGS metagenomics next-generation sequencing, FISH fluorescence in situ hybridization, MRI magnetic Resonance Imaging.

Most patients were immunocompetent, with only one patient (Patient 5) suffering from type II diabetes with diabetic ketoacidosis. Four patients reported a history of trauma preceding disease onset. Patient 1 reported a definite history of local trauma though specific details were not documented in the medical records. Patient 2 sustained a history of being stabbed by metal fragments, while Patient 3 reported bumping against unidentified objects. Patient 5 suffered skin damage caused by the adhesive tape used to secure a breathing mask. The remaining 2 patients had no documented history of trauma. However, a significant occupational exposure to tobacco was identified in Patient 4, who works in the tobacco industry and has experienced prolonged exposure to tobacco, which could increase the risk of exposure and subsequent infection [12]. The skin lesions presented initially as erythematous papules that gradually developed into indurated swelling after self-inflicted picking.

Head computed tomography (CT) scan and magnetic resonance imaging (MRI) examination in three cases (Patients 2, 4, and 6) demonstrated signs of inflammatory alterations and necrosis in the orbital, maxillofacial soft tissues and paranasal sinus, respectively. The electronic rhinoscopy examination of Patient 6 revealed nasal mucosal inflammation with extensive dry crusts that were difficult to clear. Two patients (Patients 4 and 5) have ultrasound findings of the subcutaneous soft tissues that revealed multiple areas of increased echogenicity with poorly defined margins.

**Figure 2.**
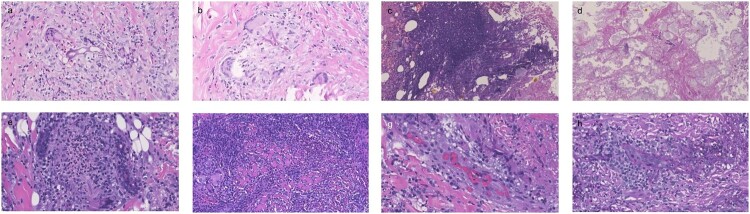
Histological findings in 6 cases of mucormycosis. (a–e, Hematoxylin and eosin stain; f, Hematoxylin and eosin stain.). (a) Large, broad, and thin-walled hyphae are visible on tissue sections. (Patient 6, magnification × 800). (b) Acutely branching, non-septate hyphae were observed in tissue sections (Patient 6, magnification × 800). (c) Necrotic debris intermixed with neutrophils, imparts a dirty blue-gray appearance (Patient 5, magnification × 400). (d) Necrotic adipocytes lack nuclei, with a large amount of pale cytoplasm with a basophilic hue due to saponification (Patient 5, magnification × 400). (e) Neutrophilic granuloma is marked by a central abscess (neutrophils) within the accumulation of macrophages (Patient 3, magnification × 400). (f) A large number of multinuclear giant cells and eosinophils were mixed with broad hyphae in the dermis and deeper tissues. (Patient 4, magnification × 400). (g) Dermal vessels showing dilated and congested (Patient 3, magnification × 800). (h) Hyphae observed without evidence of vessel wall invasion (Patient 3, magnification × 800).

A total of 6 biopsy specimens were available for histopathologic evaluation (Table 2). All lesions showed large, broad, and thin-walled hyphae in tissue sections (Figure 2(a)). These hyphae were either unseparated or oligo-segregated, and on cross-section, the hyphae gave a bubbled or vacuolated appearance. While the hyphae in most patients branched at an angle of 90 degrees, two patients were with the hyphae branching at acute angles (Figure 2(b)). The inflammatory response to the fungi varied considerably. Neutrophilic suppurative inflammations in the absence of granuloma were observed in 2 patients (Patients 2 and 4). Necrotic debris was admixed with neutrophils, imparting a dirty blue-gray appearance on low magnification (Figure 2(c)). The infection extended to adipose tissue, which resulted in significant saponification necrosis (Figure 2(d)), mimicking pancreatic panniculitis. In Patient 2, the fungal hyphae and necrosis were observed even in the orbicular muscle of the right eye. Neutrophilic granulomatous response, which consisted of an approximate equivalent mixture of neutrophils and granulomas, was seen in the other two patients (Patients 3 and 6). Typical suppurative granulomas, marked by a central abscess (abundant neutrophils) within the accumulation of macrophages, were also noted (Figure 2(e)). Distinctive eosinophilic granulomatous response was observed in the last two patients (Patients 1 and 4), both were characterized by significant infection extension to deep underlying tissues such as adipose tissue or jugomaxillary muscle (Figure 2(f)). A large number of multinuclear giant cells and eosinophils were mixed with broad hyphae in the dermis and the deep underlying tissues. In all patients in which vessels were present for evaluation (*n *= 6, 100%), dilatation and congestion of vessels in the dermis and subcutaneous tissues were observed. However, no hyphal invasion of the vessel wall (angioinvasion) was seen (Figure 2(g)). This was further confirmed by a fungus-specific stain such as PAS staining (Figure 2(h)).

**Table 2.** Histopathologic evaluation for the six patients with mucormycosis.

Patient	Histopathologic Characteristics				Fungal Characteristics		
	Main Types of Inflammation	Inflammatory Cell Types	Saponification necrosis	Morphology of hyphae	Angle of hypahe	Bubble of hyphae	Angioinvasion
1	Eosinophilic granulomatous Inflammations	Multinucleated giant cells, epithelioid cells, eosinophils, lymphocytes, plasma cells	None	Non-septate	90-degree	Yes	None
2	Neutrophilic inflammations Necrosis	Neutrophils	Yes	Non-septate or sparsely septate	90-degree 45-degree	Yes	None
3	Neutrophilic granulomatous response	Neutrophils, epithelioid cells, multinucleated giant cells, lymphocytes	None	Non-septate	90-degree	Yes	None
4	Eosinophilic granulomatous Inflammations	Multinucleated giant cells, epithelioid cells, eosinophils, lymphocytes, plasma cells	None	Non-septate	90-degree	Yes	None
5	Neutrophilic inflammations Necrosis	Neutrophils	Yes	Non-septate	90-degree	Yes	None
6	Neutrophilic granulomatous response	Neutrophils, epithelioid cells, multinucleated giant cells, lymphocytes	None	Non-septate	90-degree 45-degree	Yes	None

**Figure 3.**
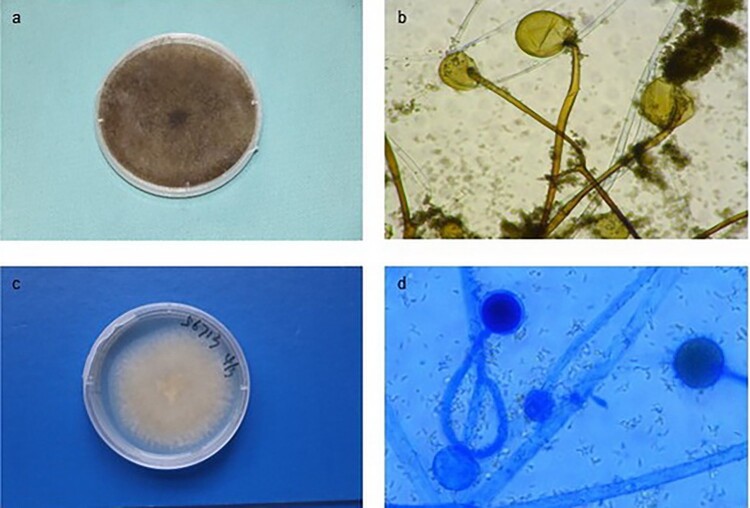
The fungal identification results of some mucormycosis patients. (a) Colonies on potato dextrose agar from Patient 4 are dense and dark gray to brown after 5 days at 28°C. (b) Microscopic examination of the cultured organisms from Patient 4 reveals spherical sporangia with visible rhizoids (magnification × 400). (c) Colonies on potato dextrose agar from Patient 6 are white to brown and velvety after 5 days at 28°C. (d) Microscopic examination of cultured organisms from Patient 6 shows spherical sporangia with visible thick-walled spores (lactophenol cotton blue stain, magnification × 400).

Fungal tissue cultures were submitted for analysis in five patients, except for Patient 1 consistent with histopathologic findings. Cultures yielded *Rhizopus* species in 3 patients (Figure 3(a,b)) and *Mucor* species in 1 patient (Figure 3(c,d)). Further species-level identification was performed using internal transcribed spacer (ITS) sequencing. For Patient 2, fungal culture yielded *Alternaria* spp., which was inconsistent with the observation of pauci-septate, broad, easily folded, and thin-walled hyphae in the histopathological examination. The potential fungal pathogens were then identified by metagenomics next-generation sequencing (mNGS) and fluorescence in situ hybridization (FISH) detection using skin lesion samples. For the five patients, the etiologic agents identified in order of frequency were *R. arrhizus* (Patient 3, 4, 5), *M. variabilis* (Patient 6), *Lichterman (L.) ramosa* (Patient 2). The details of the isolated fungal species are presented in Table 2.

Patient 5 died before commencing anti-fungi therapy. The elderly patient with multiple comorbidities, including coronary heart disease, hypertension, type II diabetes, cerebral infarction, and hypothyroidism, contracted COVID-19 and was admitted to the emergency department. Based on medical history and examination results, emergency department experts concluded that the patient died from COVID-19-induced respiratory failure. Except for Patient 5, all other patients received Amphotericin B treatment. Patient 1 and Patient 2 were treated with Amphotericin B deoxycholate, with total doses of 1 and 1.5 g, respectively. Patient 2 developed severe nephrotoxicity after taking Amphotericin B treatment and the dose was reduced and then treated with oral itraconazole after ending the Amphotericin B. Patients 3 and 6 received liposomal Amphotericin B treatment, with total doses of 0.8 and 1.3 g, respectively. Patient 4 was treated with Amphotericin B colloidal dispersion, receiving a total dose of 6.6 g. Among the five patients who received Amphotericin B therapy, all achieved recovery. However, two patients were left with severe sequelae. Among them, Patient 2 accepted an eyeball excision procedure, resulting in significant disability. In Patient 6, the infection gradually destroyed the soft palate, resulting in palatal perforation.

## Discussion

This study analyzed six cases of mucormycosis, predominantly involving the skin and mucous membranes. Definite diagnosis was established by histopathology and culture. All cases present with a chronic course of this infection. To our knowledge, reports of similar chronic cutaneous mucormycosis cases are limited, including reviews from 1991[13], 2006 [6], 2013 [14,15], and 2014 [16]. Previously published cases consistently describe a gradual onset and slow progression of symptoms, typically lacking systemic manifestations such as pain or fever. Compared to the acute cutaneous subtype, the chronic cutaneous form demonstrates a more favorable prognosis but remains associated with significant morbidity.

The underlying mechanisms for the distinct clinical presentations between acute and chronic subtypes of mucormycosis are not yet fully understood. In our cases, the microscopic analysis of affected tissues showed suppuration and granulomatous inflammation, with or without necrosis, correlating with the clinical manifestations observed in these patients. Fungal hyphae could be detected in all cases. In four cases, fungal hyphae were seen in deep tissue levels such as subcutaneous tissue, orbicular and jugomaxillary muscle, indicating extensive local invasion. However, angioinvasive features were not observed in all cases, irrespective of depth of invasion. Since angioinvasion is regarded as a critical factor driving the fast-progressing clinical course of mucormycosis [17], we hypothesize that its absence may underlie the chronic and indolent clinical progression observed in cutaneous and mucosal mucormycosis. Indeed, similar phenomena have been observed in other fungal infections, such as those caused by *Aspergillus* species. Invasive Pulmonary Aspergillosis (IPA) can be classified into acute and chronic forms. The former is an acute progressive infection that occurs in patients with conditions like organ transplantation or neutropenia and is associated with high mortality. The pathology of acute IPA is mainly due to angioinvasion, resulting in wedge-shaped infarcts or well-circumscribed nodules around blood vessels [18]. In contrast, in settings other than transplantation or neutropenia, IPA patients may present with symptoms that develop gradually over several weeks to months. Histopathologically, the chronic form of IPA is characterized by granulomatous inflammatory reactions, but absence of angioinvasion [19]. Thus, the histologic patterns observed in cutaneous mucormycosis may contribute significantly to the acute and chronic progression.

Previous studies have shown that unusual mucoralean species such as *Mucor* spp. [8], *Lichtheimia* spp. [20]*, Apophysomyces* spp. [21]*,* and *Saksenaea* spp. [22,23] have been implicated in chronic subtypes of cutaneous mucormycosis. However, *Rhizopus* species, which is the most common cause of mucormycosis, has been rarely described to cause this type of infection [16,24]. Rodríguez-Lobato et al. reported that *R. arrhizus* caused a rapidly progressive plaque with hemorrhagic vesicles and central necrosis [25]. Their retrospective review of published cases further highlighted the association of *R. arrhizus* with the acute necrotizing subtype of cutaneous mucormycosis. In our case series, the most common causative agents were *R. arrhizus* (3 cases, 50%)*,* followed by *Mucor (M.) variabilsi* (1 case, 17%) *and L. ramose* (1 case, 17%)*.* This observation is inconsistent with the distribution of mucoralean species previously reported in the chronic subtype of cutaneous mucormycosis. It confirms that, while *R. arrhizus* is primarily associated with the acute necrotizing form, it also plays a significant role in the chronic subtype, underscoring its epidemiologic and clinical significance.

Most patients in our study were immunocompetent, a finding consistent with previously reported cases of chronic cutaneous mucormycosis [16]. It indicates that the chronicity of cutaneous Mucorales infections is influenced by host factors, with immune responses playing an important role. The host's innate immune response, particularly the function of neutrophils and macrophages, is crucial in controlling *R. arrhizus* infections. These immune cells produce reactive oxygen species and cytokines that can directly combat the fungus and modulate the adaptive immune response [26]. In neutropenic patients, angioinvasion caused by Mucorales was more extensive than that in nonneutropenic patients [27]. Neutrophils from diabetic patients with hyperglycemia and diabetic ketoacidosis (DKA) retain the ability to damage fungal hyphae but show reduced responses to *R. arrhizus* chemotactic factors [28]. Macrophages from diabetic or corticosteroid-treated mice fail to inhibit spore germination [29]. Thus, it seems that differences in the outcome of acute versus chronic cutaneous mucormycosis may be related to the host's immune status. However, in our study, there was still one patient with a history of diabetes who did not show evidence of fungal vascular invasion. Indeed, similar indolent courses of cutaneous mucormycosis have been described for diabetic individuals. In a case series reported by Arnáiz-García et al, there was one 72-year-old man with diabetes mellitus who developed an ulcerous dark-yellow nodular lesion on the right leg 6 months before [30]. Similarly, it was also reported in a 54-year-old woman patient with diabetic ketoacidosis [31]. The latter case presented with an ulcerated lesion with purulent discharge on the scalp about one year before. These observations suggest that the host’s immune status may not be the sole factor in differentiating acute and chronic forms of cutaneous mucormycosis.

The interaction between Mucorales and epithelial cells remains poorly understood. The Mucorales express spore coat homolog (CotH) proteins, which bind to the receptor glucose-regulator protein 78 (GRP78) on the endothelial cells [32]. Since the number of CotH genes varies among Mucorales species, the angioinvasive ability may depend on the number of CotH copies expressed [33]. In addition, endothelial damage is mediated by the secondary metabolites produced by the Mucorales, which act as toxins [27]. It is possible that a ricin-like toxin, mucoricin, enhances angioinvasion and tissue destruction. Nevertheless, it is likely that host–pathogen interactions, rather than fungal species- or host-specific factors, play a more important role in determining angioinvasive nature. For this angioinvasion process to occur, the fungus must first come into contact with endothelial cells, which are typically separated by basement membranes containing laminin and collagen IV. Tissue damage induced by external forces or wounds resulting in conditions such as diabetes or chemotherapy exposes these extracellular proteins, which then serve as adhesion points for fungal spores [34]. Furthermore, abnormalities in iron metabolism and hyperglycemia in diabetes patients facilitate the interaction between CotH3-expressing Mucorales and the endothelial GRP78 receptor, promoting angioinvasive fungal growth [35]. Besides, transcriptomic analyses of endothelial cells interacting with *Rhizopus* or *Mucor* have shown upregulation of the platelet-derived growth factor receptor B (PDGFRB), with its inhibition reducing the cell damage caused by Mucorales [33]. Thus, it can be inferred that the interaction between Mucorales and the host plays a crucial role in vascular invasion in mucormycosis.

Two of the six patients in this study had a history of trauma, including stabbing by metal fragments of motor and bumps against unidentified objects. This observation suggests that pathogen inoculation following trauma is a common mode of chronic cutaneous mucormycosis. Additionally, occupational exposure to tobacco has been identified as a possible novel predisposing factor for *R. arrhizus* infection in our case series. In China, *R. arrhizus* causes tobacco pole rot during the tobacco flue-curing [36]. Another environmental mycology study found that the counts of fungal colonies such as *R. nigricans* and other species, were significantly higher in tobacco workshops than control environments [37]. Therefore, occupational exposure of tobacco producers could increase the risk of exposure. Undetected trauma to the skin could lead to a local breakdown, allowing fungal spores to inoculate the dermis or subcutaneous tissue, thereby resulting in cutaneous mucormycosis. There was one patient without obvious trauma histories but with significant MRI alterations of the sinus’s soft tissues. We propose that inhalation inoculation of the organism to the paranasal sinus is the major portal of entry. Contiguous extension to the palate may lead to necrotic ulcer. Intraorally, the upper palate is typically involved due to its anatomical proximity to the nasal cavity and paranasal sinuses, facilitating the extension of the infection.

To date, there is a lack of awareness in the dermatology literature regarding the chronic subtype of cutaneous mucormycosis. Unlike the acute subtype, the lesions of the chronic subtype exhibit a greater variability in appearance, ranging from erythematous superficial plaque, deep nodules, and indurated swelling, to necrotic ulcers. This less-known clinical presentation of the chronic subtype differs from the acute subtype, as depicted in Table 3. The clinical differential diagnosis of chronic cutaneous mucormycosis includes leishmaniasis, sporotrichosis, lupus vulgaris, sarcoidosis, Wegener granulomatosis, necrobiosis lipoidica, and natural killer and T-cell lymphomas. Compared with the acute type, the differential diagnosis of the chronic subtype of cutaneous mucormycosis is substantially broader, and early diagnosis is challenging due to the nonspecific nature of its clinical features and symptoms.

**Table 3.** Clinical, histopathological and prognosis differences between acute and chronic mucormycosis (proposed).

	Acute cutaneous mucormycosis	Chronic cutaneous mucormycosis
Onset	Acute	Chronic
Progression	Rapid	Slow
Cellular immunity	Usually impaired	Usually intact
Clinical features	Targetoid plaques Bull’s-eye infarctions Ecchymotic lesions Rapid progressive necrosis with eschar formation	Superficial or infiltrated plaques Deep nodules Slowly progressive necrotic ulcers Indurated swelling
Histopathology	Neutrophilic necrotic inflammations Angioinvasion (+)	Neutrophilic inflammations ± Necrosis Neutrophilic granulomatous response ± Necrosis Eosinophilic granulomatous Inflammations Angioinvasion (−)
Prognosis	Poor can lead to mortality	Good, usually no mortality but causes significant morbidity

Because the diagnosis is usually not suspected clinically, pathologists play a pivotal role in facilitating the timely identification of mucormycosis. This disease has distinctive histologic features, usually with broad unseparated banded hyphae at a 90-degree angle [17]. However, our study found that the hyphae branching can also occur at 45 degrees. We assume that it might be due to interstitial pressures exerted on the fungi by the tissue and alterations in tissue architecture during processing. Thus, the wider and ribbon-like nature of the hyphae are more reliable distinguishing characteristic than the angle of branching. Previous studies have reported host tissue reactions to include suppurative inflammation and granuloma formation. Eosinophil infiltration was uncommon and could help to differentiate cutaneous mucormycosis from cutaneous entomophthoramycosis. Consistent with these findings, we observed similar histopathologic features in our 6 patients with chronic cutaneous and mucosal mucormycosis. The observed changes include granulomatous inflammation, necrosis, multinucleated giant cells and neutrophil infiltrates. When subcutaneous adipose tissue was affected, ghost adipocytes were observed, which is very similar to the pathological manifestations of pancreatic panniculitis [38]. In contrast to previous studies, we observed prominent eosinophilic infiltration in 2 of 6 patients although the Splendore-Höeppli phenomenon around the hyphae was absent. These findings differ from earlier reports on cutaneous mucormycosis. Mendoza et al considered that subcutaneous zygomycosis caused by mucoralean species does not trigger an eosinophilic infiltration in the infected area, and this feature has been used to differentiate infections caused by members of the Mucorales and Entomophthoromycota [39]. Our findings may reflect the complex interaction between Mucorales and the host. Some species of Mucorales appear capable of eliciting a local inflammatory response characterized by eosinophilic infiltration; however, the pathogens seem to survive this initial attack and subsequently spread to adjacent tissues.

Differentiation between these subgroups has prognostic implications. According to the literature, the mortality rate in localized cases is 4% to 10%, for deep lesions it increases to 26%–29%, and in those with dissemination from a skin focus it may be as high as 83%–94% [4]. In our study, the prognosis of chronic cutaneous zygomycosis was notably better compared to other clinical forms of the disease. As most patients recovered after amphotericin B alone, it seems that comprehensive removal of all infected tissue may not be a prerequisite for successful treatment of chronic cutaneous and mucosal mucormycosis, whereas in the acute subtype remaining infected tissue will result in the relentless spread of the fungal infection into adjacent tissue and eventually treatment failure.

While our study provides valuable insights into the clinical and pathological characteristics of chronic cutaneous and mucosal mucormycosis, we acknowledge that the sample size of six cases is modest. This limitation is due to the rarity of the condition, which restricts the availability of cases for analysis. Consequently, larger, multi-center studies with more extensive patient cohorts are necessary to validate our results and further investigate the broader implications of chronic mucormycosis across diverse populations and clinical settings.

## Conclusion

Chronic cutaneous mucormycosis represents a distinct subtype of Mucorales-related cutaneous infections. Unlike the acute subtype, it displays a broader spectrum of clinical manifestations, is often associated with foreign trauma, and typically demonstrates a more favorable prognosis while still posing a risk for significant disability. The absence of fungal angioinvasion is likely the key factor underlying its indolent progression. The immune status of the host could not be a distinguishing factor between the two forms. There may be no difference in causative species between chronic and acute cases, as *R. arrhizus* was the most frequently isolated species in our case series. Dermatologists should maintain a high level of awareness for this rare condition when evaluating unexplained granulomatous lesions.

## Ethical statement

This study was approved by the Clinical Research Ethics Committee of the Xijing Hospital, Fourth Military Medical University. The approval number is KY20172030-1. We obtained blood samples from the patients and their parents, after obtaining informed consent. The patients permitted to publish their images and medical information.
